# RNA disruption indicates CHOP therapy efficacy in canine lymphoma

**DOI:** 10.1186/s12917-019-2189-x

**Published:** 2019-12-16

**Authors:** Amadeo M. Parissenti, Laura B. Pritzker, Baoqing Guo, Rashmi Narendrula, Shirly Xiaohui Wang, Lin Laura Lin, Jingchun Pei, Karolina Skowronski, Dorothee Bienzle, J. Paul Woods, Kenneth P. H. Pritzker, Brenda L. Coomber

**Affiliations:** 1 Departments of Biology, Chemistry and Biochemistry, Laurentian University, 935 Ramsey Lake Rd., Sudbury, ON P3E 2C6 Canada; 2 Rna Diagnostics, 21 St. Clair Avenue East, Suite 701, Toronto, ON M4T 1L9 Canada; 3 Rna Diagnostics, c/o Health Sciences North Research Institute, 2nd Floor North, 56 Walford Road, Sudbury, ON P3E 2H3 Canada; 4 Department of Clinical Studies, Ontario Veterinary College, University of Guelph, 50 Stone Road East, Guelph, ON N1G 2W1 Canada; 5 Department of Pathobiology, Ontario Veterinary College, University of Guelph, 50 Stone Road East, Guelph, ON N1G 2W1 Canada; 6 Department of Biomedical Sciences, Ontario Veterinary College, University of Guelph, 50 Stone Road East, Guelph, ON N1G 2W1 Canada

**Keywords:** Chemotherapy treatment response, RNA analysis

## Abstract

**Background:**

Assessment of the efficacy of a multi-agent chemotherapy protocol in which cyclophosphamide, doxorubicin, vincristine and prednisone (CHOP) are administered in canine lymphoma is generally performed by physical measurement of lymph node diameter. However, no consistent correlation has been made with prognostic indicators and the length or absence of clinical remission based on lymph node size. RNA disruption measured mid-therapy has been correlated with increased disease-free survival in recent studies of human cancer and was assessed in this study of canine lymphoma patients. Fine needle aspirate samples were taken before treatment and at weeks 3, 6, and 11 of CHOP therapy. RNA was isolated from these samples and assessed using an Agilent Bioanalyzer. RNA disruption assay (RDA) analysis was performed on the data from the resulting electropherograms.

**Results:**

An increased RNA disruption index (RDI) score was significantly associated with improved progression-free survival.

**Conclusions:**

Predicting the risk of early relapse during chemotherapy could benefit veterinary patients by reducing ineffective treatment and could allow veterinary oncologists to switch earlier to a more effective drug regimen.

## Background

Canine high grade nodal lymphoma, the most common haematopoietic neoplasm in dogs [1], is routinely treated with a multi-agent chemotherapy protocol in which cyclophosphamide, doxorubicin, vincristine and prednisone (CHOP) are administered on a weekly or biweekly basis over four months [2]. Remission rates from CHOP therapy range from 73 to 92% [2] and result in a median first remission duration of 5–12 months [3], with wide variation around the median. Response to therapy is assessed by physical measurement of lymph node size; complete clinical remission is defined as the lymph nodes returning to normal size [4]. Many factors including age, sex, breed, neutropenia and World Health Organization (WHO) stage have been proposed to have an impact on prognosis but only tumour stage, grade, immunophenotype and clinical response to chemotherapy have been well established to have an effect on patient survival [5]. B cell lymphomas have been found to have a better prognosis than most T cell lymphomas [5]; however, specific immunotypes in T cell lymphoma are associated with improved survival and a longer progression free interval [6].

A number of studies have explored methods to predict outcome in canine lymphoma including refining diagnostic staging methods using cytology and sonography to evaluate the liver and spleen [7], measurement of chemotherapy-induced neutropenia [8], assessment of the relative expression levels of BCL2 and MYC [9], the use of the modified Glasgow Prognostic Score [10]), the immunosignature at diagnosis [11], the assessment of Ki67 and its correlation with mitotic index [12] and flow cytometric characterization of S-phase fraction and ploidy in lymph node aspirates [13]. To date, no useful prognostic indicators have been established that consistently correlate with either the length or lack of remission.

The desire to identify prior to or early during treatment those cancer patients that will derive a survival benefit from chemotherapy is common to both veterinary and human medicine. Approximately 20% of breast cancer patients treated with neoadjuvant chemotherapy (chemotherapy prior to surgery) achieve a complete response as assessed by histopathology and derive a survival benefit from chemotherapy [14]; yet, almost all patients endure the toxic side effects of treatment [15, 16]. The use of RNA integrity was first identified as a prognostic marker in a human breast cancer clinical trial in which patients undergoing treatment with neoadjuvant chemotherapy had core needle-biopsy samples taken pre-, mid- and post-therapy. RNA was isolated from these samples and mid-therapy samples were found to have varying degrees of RNA integrity. Low biopsy RNA concentration and low RNA integrity, as assessed by the RNA integrity number (RIN), were associated with complete response by histopathology (pCR) [17]. Unique rRNA banding patterns were identified in RNA electropherograms of patient samples treated with chemotherapy, and this effect was subsequently termed “RNA disruption” [18]. Analysis of the rRNA fragmentation patterns using the RNA electropherograms led to the creation of an RNA Disruption Index (RDI), which specifically quantifies the degree of rRNA fragmentation in a given sample. High tumour RDI values were subsequently found to be associated not only with pCR but also with improved disease-free survival [18]. Subsequent studies showed that RNA disruption could be induced in vitro by treating cancer cells with a variety of chemotherapy agents including docetaxel and carboplatin [19]. RNA disruption also occurred in samples from breast cancer patients treated with zoledronic acid [20] and in human prostate tumour xenografts treated with docetaxel or a docetaxel/relaxin hormone receptor agonist (AT-001) combination [21]. An additional study found a strong correlation between RNA disruption after one cycle of docetaxel, carboplatin, trastuzumab ± lapatinib and pCR in a small group of Her2+ breast cancer patients [22].

In this study, we investigated RNA disruption as a predictor of remission duration in canine lymphoma to understand if RNA disruption occurred and had clinical utility outside of breast cancer; tumour RNA disruption in human lymphoma patients has not yet been investigated. RNA disruption was measured in fine needle aspirate (FNA) samples of tumour tissue obtained at four time points (0, 14, 28 and 56 days) during CHOP therapy. This protocol permitted assessment of how early in treatment RNA disruption may be measured and how tumour RDI values change over time within a given patient. We then analysed RDI values in relation to clinical response and the progression free interval.

## Results

Forty-one dogs fulfilled the inclusion criteria. Assessment of the clinical response during CHOP therapy found that 31 dogs achieved a CR for at least one timepoint, 4 dogs had only aPR, 1 dog had a PR which was followed by PD, 4 dogs had only PD, and 1 dog had only SD. Five dogs that initially had a CR relapsed during the course of CHOP therapy; the breakdown of all dogs by clinical response during treatment is shown in Table 1. Five dogs were lost to follow up and could not be included in the assessment of PFS or in the assessment of timing of relapse as a response to CHOP therapy.

**Table 1:** Clinical Response of dogs as they progressed through CHOP therapy

Clinical Response to CHOP	Number of Dogs
CR (At all timepoints)	26
CR (Followed by PD)	5
PR (At all timepoints)	4
PR (Followed by PD)	1
SD	1
PD (At all timepoints)	4

Summary of clinical response assessment of all dogs at weeks 2, 5, and 10 during CHOP therapy grouped by either consistent response throughout therapy or change in response during therapy.

RNA concentrations and RDI values were determined for FNA samples taken pre-therapy and at approximate timepoints of 14, 35 and 70 days. A decrease in biopsy RNA concentration was found upon chemotherapy treatment which corresponded with clinical response measured at the same time point. A linear mixed effects model was fitted to the data using RNA concentration and clinical response as covariates. The median RNA concentration was about 10 fold higher from samples taken when the clinical response was determined to be progressive disease as opposed to those taken when there was a complete response (217 ng/μl versus 26 ng/μl respectively; 95% confidence interval [50.8, 398.2])) (*p*-value = 0.04; likelihood ratio test).

The adequacy of the canine lymphoma samples was then assessed for specific ranges of sample acquisition times, as defined in Materials and Methods. As shown in Table 2, sample adequacy was dependent upon the duration of treatment; at 0 weeks 5% of samples were inadequate which became 22% after 2–3 weeks and 50% after 4–6 weeks. The inability to determine the morphologic diagnosis of “Lymphoma” by cytology also increased with duration of treatment from 15% “No Diagnosis” samples at 0 weeks to 37% at 2–3 weeks and 50% at 4–6 weeks. Most of the samples (79%) which were designated as “inadequate” for RDA had corresponding samples taken for cytology assessment that were also designated “No Diagnosis” based on the absence of nucleated cells. Differences within the samples may have resulted in the disparities between RDA and cytology results; however, of the remaining “inadequate” samples which were assessable by cytology 13% had very low cellularity (1–24%). This likely contributed to a lack of analyzable RNA in the FNA assessed by RDA.

**Table 2:** RDA sample adequacy and morphologic diagnosis of FNA samples acquired during CHOP chemotherapy administration

Time Sample Acquired	Number of RDA Adequate Samples	Number of RDA Inadequate Samples	Total Samples for RDA	% Inadequate Samples	Total Samples for Cytology	Number of “No Diagnosis”Samples	% “No Diagnosis”Samples
0	39	2	41	5	40	6	15
2–3 weeks	25	7	32	22	30	11	37
4–6 weeks	16	16	32	50	32	16	50
7+ weeks	24	15	39	38	36	16	44

The RDA sample adequacy for samples taken within specific windows of CHOP therapy administration (2–3 weeks, 4–6 weeks and 7+ weeks) were assessed and compared with the results of cytology measured in parallel samples acquired at identical timepoints.

We then examined if there was a significant relationship between RDI value and the neoplastic cell score as determined by cytology. Two weighted linear regression models were fitted to the observed data (one for RDI and one for RNA concentration), and the hypothesis tests for the relationship between RDI/RNA concentration and cellularity were conducted. A higher proportion of neoplastic cells was significantly associated with lower RDI values (*p*-value = 0.008) and higher RNA concentration (*p*-value = 0.05). Average RDI value and average RNA concentration for each neoplastic cell score (NCS) is shown in Table 3. Average RDI scores increase as the neoplastic cell score decreases (0.6 at NCS of 3 to 1.0 at NCS of 2 and 1.8 at 1). The FNA samples that had a neoplastic cell score of “0” had an average RDI value of 0.6 (*n* = 5); however, the absence of neoplastic cells in these samples was noted as “No Diagnosis”. These samples also had overall cellularity scores of “0”, reflecting a complete absence of all cells. No conclusions can therefore be made on RDI values and a neoplastic score of “0” from this dataset.

**Table 3:** Relationship between neoplastic cell score and RDI value or RNA concentration

Neoplastic Cell Score^a^	n	Average RDI	Std Dev RDI	Range RDI	Average RNA Concentration (ng/uL)	Std Dev RNA Concentration	Range RNA Concentration (ng/uL)
0	5	0.6	0.3	0.3–1.0	74	101	5.0–253
1	16	1.8	2.0	0.2–7.9	79	137	3.8–495
2	7	1.0	0.8	0.4–2.9	178	227	11.9–601
3	67	0.6	0.6	0.1–3.3	315	417	0.6–2726

The neoplastic cell score was assessed by cytology using the following criteria: Proportion of cells assessed as neoplastic; 0 (0%), 1(1–24%), 2(25–49%), 3(> 50%). The RDI values and RNA concentrations were then grouped by neoplastic cell score and the average value, standard deviation and the range of values were generated.

**Fig. 1:**
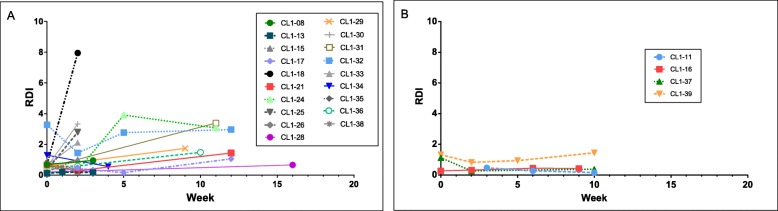
RNA disruption during CHOP therapy for dogs separated by immunophenotype. RNA disruption during CHOP therapy for dogs separated by immunophenotype A) dogs that had B cell lymphoma (*n* = 19) and B) dogs that had T cell lymphoma (*n* = 4). Five dogs were found to have inadequate samples at all time-points and were therefore not included; 13 dogs did not have their tumour immunophenotype determined. Each line represents an individual animal

Immunophenotype was determined for 28 out of the 41 dogs in the study (B cell lymphoma *n* = 22, T cell lymphoma *n* = 6) and the generated RDI values for all B cell lymphomas and all T cell lymphomas during CHOP therapy were graphed (Fig. 1a and b respectively). Although the tumour RDI values for dogs with B cell lymphoma were higher than for dogs with T cell lymphoma, the differences were not statistically significant due to the small number of dogs with T cell lymphoma having assessable RDI values (*n* = 4). As expected, the dogs with T cell lymphoma in this study were 5.7 times more likely (95% confidence interval [1.9, 16.9) to have disease relapse compared to dogs within the B cell group (Wald-test *p*-value = 0.002).

**Fig. 2:**
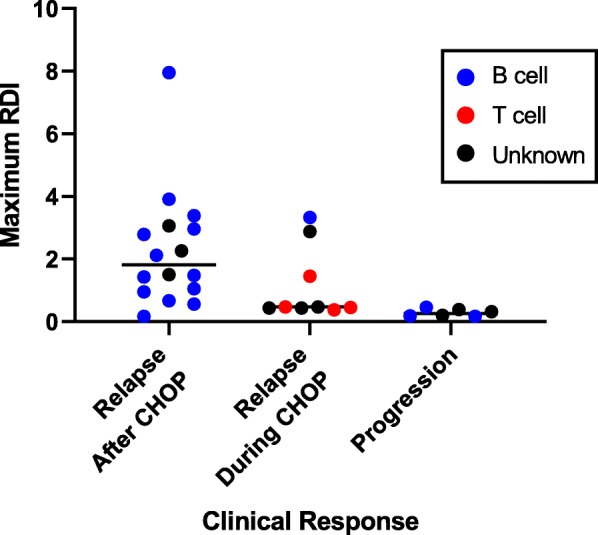
Maximum RDI Values and Response to CHOP Therapy. Maximum RDI values were determined for each dog for all FNA samples taken during therapy. RDI values were grouped by relapse status; relapsed after CHOP therapy, relapsed during CHOP therapy, or progressive disease. RDI values were higher in dogs that responded to treatment (and had a relapse after the completion of CHOP therapy) compared with dogs that had a relapse during therapy (Mann-Whitney test *p* = .04)

To determine if RDI values correlated with clinical response, the maximum RDI value was determined for each dog and these values were then grouped by response to CHOP therapy: dogs that relapsed after the completion of CHOP therapy (*n* = 16), dogs that had a CR and subsequently relapsed during CHOP therapy (*n* = 9), and dogs that progressed during CHOP therapy (*n* = 6) ((Fig. 2). Ten dogs were excluded from this analysis: six had inadequate samples at all timepoints (3 B cell, 2 T cell and 1 Unknown immunophenotype), while four were lost to follow up and did not have a time to relapse. Tumour RDI values were higher in dogs that relapsed after CHOP chemotherapy (median RDI value 1.8) than in dogs which had a relapse during CHOP therapy (median RDI value 0.5). A Mann-Whitney test was employed and identified a significant association between maximum RDI values and the timing of a relapse (during chemotherapy or after completion of chemotherapy); *p* = 0.04.

**Fig. 3:**
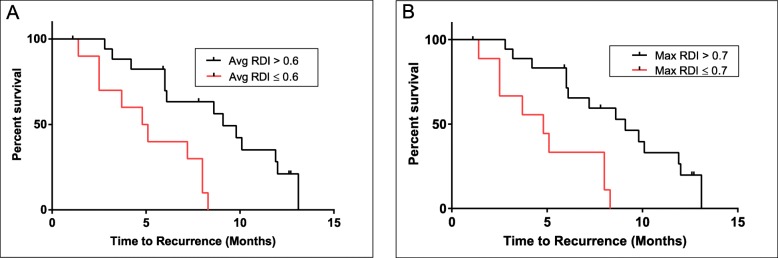
Kaplan-Meier plots of Progression-Free Survival. Kaplan-Meier plots depicting differences in PFS over time between patients with A) average RDI values > 0.6 and ≤ 0.6 and B) maximum RDI values > 0.7 and ≤ 0.7. Hazard ratios associated with the RDI values are listed in the plots. Differences between survival curves are statistically significant (log-rank test *p* = 0.006 for average RDI and log rank test p = 0.006 for maximum RDI)

A Cox proportional hazards model was fit to the data to determine whether or not RDI was associated with PFS. Both average RDI and maximum RDI were assessed for their ability to predict relapse. An average tumour RDI of < 0.6 was associated with high-risk for relapse, as these patients had 4.3 times the risk experiencing a relapse compared to the tumours of patients with RDI values > 0.6 (95% confidence interval [1.5, 12.0]). The relative risk of relapse was lower for dogs with higher maximum tumour RDI compared to dogs with lower maximum tumour RDI. Dogs with a maximum RDI ≤0.7 had 4.1 times the risk of experiencing a relapse compared to dogs with maximum tumour RDI > 0.7 (95% confidence interval of [1.5, 11]). These RDI cut points of 0.6 and 0.7 were then used to generate the Kaplan-Meier plots shown in Fig. 3.

The Kaplan-Meier plot in Fig. 3a illustrates that PFS is increased for dogs that have an average RDI > 0.6. The median PFS time for dogs with an average RDI > 0.6 was 9.1 months compared with 4.9 months for RDI ≤ 0.6; the difference in these two curves was statistically significant (log-rank test *p* = 0.006). Similarly, as shown in Fig. 3b, PFS is increased for dogs that have a maximum RDI > 0.7. The median PFS time for dogs with a maximum RDI > 0.7 was 8.9 months compared with 4.8 months for RDI ≤ 0.7; these were also statistically significant differences (log-rank test p = 0.006).

The utility of RDA in providing additional information to predict PFS was examined by comparing clinical response during CHOP therapy (standard of care) to the RDI values obtained during therapy. Clinical response was effective in predicting PFS for most dogs undergoing CHOP therapy, especially for those that responded well to treatment and those that showed no response. However, RDI values may be a useful discriminator in some cases. We identified three dogs that had complete clinical responses during treatment and yet relapsed before 6 months. The RDI values for these dogs fell below 1.4 at all time points and below 0.9 at time points up to 6 weeks, which included early-treatment and mid-treatment values (Table 4).

**Table 4:** Dogs having a complete clinical response followed by a relapse before 6 months

Dog	Phenotype	Relapse (months)	Time (weeks) from Start of Treatment	Clinical Response	RDI Value
CL1–14	Unknown	5.1	2 3 6 10	Not recorded CR CR CR	0.4 0.3 n/a n/a
CL1–37	T cell	3.7	2 4 10	CR CR All WNL except pop mildly enlarged L 2 and R 1.5	0.3 n/a 0.4
CL1–39	T cell	3.2	2 5 10	CR CR CR	0.8 0.9 1.4

## Discussion

There is considerable interest in investigating methods to monitor the response to CHOP therapy in canine lymphoma which would allow the prediction of early relapse before the reappearance of clinical signs. Monitoring the effectiveness of therapy could lead to modification of chemotherapy protocols and improved patient outcome. Molecular imaging techniques such as positron emission tomography using 3′-deoxy-3′[^18^F] fluorothymidine [23] or 2-deoxy-2-[^18^F]fluoro-D-glucose [24] have been explored in small pilot studies but such approaches are quite costly and limited in their availability in veterinary medicine. Minimal residual disease in canine lymphoma has been assessed using several methods including RT-qPCR to evaluate antigen receptor gene rearrangements [25] and rearranged IgH and TCRχ genes [26]; these have been suggested to be a useful marker for treatment efficacy. Pre-treatment clinical features such as serum concentrations of C-reactive protein and albumin [10] have been measured in order to predict outcome of dogs with canine lymphoma. Biodynamic digital holography has been used to investigate drug response in ex vivo tumour biopsies and to identify biomarker signatures associated with chemoresistance [27].

This study investigated RNA disruption in biopsy samples from dogs during CHOP therapy to assess RDI as a predictor of remission duration in canine lymphoma. Samples were taken at four time points (0, 14, 35 and 70 days) to evaluate how early in treatment RNA disruption may be measured and how tumour RDI values change over time within a given patient. RNA disruption, as measured by increases in tumour RDI value, occurred during CHOP therapy in FNA samples obtained from dogs with canine lymphoma. The increase in tumour RDI values from baseline can occur as early as two weeks after initiation of chemotherapy. It is often associated with ‘inadequate’ subsequent samples and is reflective of increased PFS. Although this pilot study was not powered to show statistically significant correlations at specific early time points, the clinical utility of RDA is in the assessment of RNA disruption early in therapy to allow veterinarians and owners to make decisions relating to the continuation or modification of chemotherapy.

In this present study, adequacy rates for acquired samples were calculated at each time point, as RNA concentrations in a human breast cancer trial were found to be decreased at mid-therapy compared to pre-therapy samples [18]. A subsequent study in similar breast cancer patients found that each cycle of chemotherapy resulted in decreased RNA concentrations [28] and in some cases, RNA concentrations decreased such that samples became ‘inadequate’ for RDA analysis. Thus, early sampling for RDA analysis seems essential not only to minimize exposure to ineffective treatments in non-responding patients, but also to have the greatest chance of obtaining a sample that will yield an informative RDI value. Early sampling also has high clinical utility as it allows clinicians to make decisions on making a switch to a more efficacious treatment.

It is also likely that the extent of RNA disruption is relevant for outcome prediction. Narendrula et al. provided evidence that a threshold amount of RNA disruption was required for cell death [19]. Although there were too few data points in the present study to generate reliable cut-points identifying non-viable cells, our previous work in a human breast cancer trial established three zones of RDI values, which corresponded to statistically significant differences in disease-free survival among patients [18]. In this current canine study, the extent of RNA disruption as assessed by the range of RDI values in on-treatment biopsies was considerably decreased. A number of factors may affect these differences in the extent of RNA disruption, including the disease type, time of biopsy acquisition, the drug regimen, drug doses and sample type (FNA vs core biopsy).

Lymph nodes often have a number of non-neoplastic cells present which may not be susceptible to RNA disruption during chemotherapy. In this study samples with neoplastic cell scores of 2 and 1 had increased RDI values compared to samples with neoplastic cell scores of 3, suggesting that samples from tumours which are most actively responding to treatment have a larger amount of RNA disruption compared with samples from tumours that were not responding. The samples in this study with a complete absence of neoplastic cells were considered to be non-diagnostic and were devoid of all cellularity; no conclusions could be made on RDI values and neoplastic cell score of 0.

While CHOP therapy is a standard protocol for canine B cell lymphoma, it has been reported to be less effective against T cell lymphoma [1]. The analysis of RNA disruption reported here supports this differential response to CHOP therapy as the samples obtained from dogs with T cell lymphoma had lower RDI values compared to dogs with B cell lymphoma. However, the sample size for T cell lymphoma was too small to permit assessment of the statistical significance of this finding.

The mechanism of RNA disruption is not fully understood but initial studies have indicated that a variety of chemotherapy drugs induce RNA disruption in vitro including paclitaxel, docetaxel, carboplatin, cisplatin, etoposide, vincristine, irinotecan and doxorubicin [19]*.* In vitro, RNA disruption was found to be initiated concurrently with apoptosis versus necrosis as indicated by Annexin-V and propidium iodide uptake measured by flow cytometry. Inhibition of caspase-3 activity significantly reduced RNA disruption in that study [19]. Additionally, RNA cleavage is associated with autophagy and apoptosis mediated by caspase-3 activation [29, 30]. The interaction of eukaryotic initiation factor 3 subunit f (elF3f) and heterogeneous nuclear ribonucleoprotein (hnRNP) K has also been identified as key to maintaining RNA stability [31]. Cells under stress, such as during chemotherapy treatment, have increased elF3f, which induces both a strong reduction in the binding of hnRNP K to the 28S rRNA as well as increased RNA degradation [31].

The treatment of canine lymphoma has evolved to include the dissection of subtypes [6], the expansion of multi-agent chemotherapy, and the development of targeted drugs and stage-adapted therapies [1, 5]. Knowledge gained from canine comparative oncology studies including those in canine lymphoma can augment knowledge of human disease; naturally occurring canine tumours have been explored as a better model to assess therapeutic response than xenograft mouse models [32]. Many similarities between human non-Hodgkin’s lymphoma and canine lymphoma have been identified [33, 34]; however, species differences have also been noted in tumour responses [35] and some methods which are prognostic in human disease have been shown not to be effective in dogs [9]. A number of recent studies have been carried out using canine lymphoma as a model system to evaluate novel therapies in Phase I trials [36] and examine chemoresistance [27]. This RDA study provides evidence that during chemotherapy treatment, canine lymphoma also undergoes RNA disruption similar to other cancers such as breast cancer [18, 22] and indicates that the extent of RNA disruption during treatment can be used to predict clinical response and outcome after chemotherapy.

## Conclusions

Although this pilot study was limited in scope due to the small number of cases, RNA disruption was identified as a novel approach which correlated with PFS and identified dogs at risk for early relapse. The assessment of RNA disruption during the treatment of this disease is an additional assessment tool, which could contribute to improved treatment and prognoses for dogs as well as reduced costs for owners and insurers.

## Methods

Dogs were diagnosed with multicentric nodal lymphoma by cytological assessment of lymph node aspirates [37] at the Ontario Veterinary College Mona Campbell Centre for Animal Cancer, University of Guelph. Dogs who had not received prior treatment other than a single injection of prednisone were eligible for this study (*n* = 41); prednisone treatment status was known for only 31 dogs (Untreated *n* = 26, Treated *n* = 5). No breed, sex or age restrictions were in place, but dogs with concurrent or prior neoplasia (including prior lymphoma) were excluded. Dogs received standard CHOP therapy) [2] (details in Additional file 1: Table A.1). A group of four dogs was switched to a modified protocol of CHOP therapy due to “poor tolerance” as assessed by the clinicians (criteria included anorexia, vomiting, diarrhea or myelosuppression causing treatment delays). This modified regimen included the addition of L-asparginase to CHOP (a single dose at 400 IU/kg to a max of 10,000 U/dog); these dogs were included in this analysis. Additional file 2: Table A.2 lists the four dogs given the modified CHOP regimen and the weeks when L-asparginase was given. As well, dose reductions of 10% were given to 13 of 41 dogs due to neutropenia; if neutropenia or clinical signs resolved, drug doses were gradually increased back to standard dose. Standard of care follow-up of canine lymphoma patients was done by monitoring clinical response through the measurement of lymph node size using calipers [4] at day 0 (prior to chemotherapy) and then for a minimum of 6 months. Using VCOG criteria: complete response (CR) was defined as all palpable lymph nodes returning to normal size, while partial response (PR) was defined as all palpably enlarged lymph nodes having a > 30% reduction in the mean sum longest diameter. Progressive disease (PD) was considered to be the presence of any new enlarged lymph nodes and/or a > 20% increase in the mean sum longest diameter of palpably enlarged lymph nodes, while stable disease (SD) was failure to obtain CR, PR, or PD [38]. Relapse was diagnosed by a recurrence of enlarged lymph nodes after a CR and was confirmed by cytology. Following the conclusion of the study period, dogs continued to receive appropriate standard of care treatment as determined by discussions between owners and veterinarians.

Fine needle aspirate samples were collected (by multiple observers) from each dog on day 0 (prior to receiving chemotherapy), and at the end of the first, second and third round of CHOP therapy (approximately 14, 35 and 70 days after the start of treatment, respectively). Thus, a maximum of four FNA samples were obtained per patient. Dogs with lymphoma that relapsed during CHOP therapy or with lymphoma that did not have a clinical response were moved onto modified or alternate regimens (including CCNU (lomustine), MOPP (combination of mechlorethamine, vincristine, procarbazine, predinsone) or DMAC (combination of dexamethasone, melphalan, actinomycin D and cytosine arabinoside) as per standard of care at the owner’s discretion. FNAs were collected from enlarged lymph nodes using multiple passes of 22 gauge sterile needles connected to a 6 mL syringe; whenever possible, the same lymph node was sampled on each occasion. Aspirates were used to generate cytology slides and the remainder of sample was expressed into sterile collection vials containing 1.0 mL of RNAprotect Cell Reagent (Qiagen) in order to prevent further degradation of the RNA within the aspirate. Immunophenotype was determined by flow cytometry according to protocols described in Deravi et al., 2017 [6]. Cytology slides were Wright-stained to determine the percentage of cancer cells in samples. Samples in RNAprotect Cell Reagent were stored at room temperature overnight and then at -80 °C prior to shipment. Samples were shipped at 4 °C to the RnaDx laboratory (Sudbury, ON) by overnight courier. Samples were stored for 9–457 days at -80 °C until RNA isolation.

RNA was isolated from samples using miRNeasy kits™ from Qiagen, Inc., and an aliquot of each sample was loaded onto a RNA Nanochip™ (Caliper Technologies) and component RNAs resolved by capillary electrophoresis on an Agilent 2100 Bioanalyzer. The resulting electropherogram data were used to determine sample adequacy, based on the presence of RNA peaks distinguishable from baseline. Electropherograms were then analyzed using proprietary algorithms to quantify RNA disruption [the RNA disruption index (RDI)] and RNA concentration [18] in adequate samples. Average RDI values (when determined) were calculated omitting the pre-therapy score.

Cytology slides were reviewed by a veterinary pathologist (DB) for the purpose of quantifying clinical response through estimation of tumour cellularity. Criteria evaluated by the pathologist for each sample included cellularity, cell size, cell integrity, neoplastic cells, anisocytosis, and the presence of red blood cells. Using these data, a morphologic diagnosis was made of either “Lymphoma” or “No Diagnosis” (due to insufficient nucleated cells). The proportion of neoplastic cells in cytosmears (‘neoplastic cell score’) was scored as 0 (0%), 1 (1–24%), 2 (25–50%), 3 (> 50%). All analyses were carried out in a blinded fashion; at the conclusion of the study RDI values and clinical data (including cytology and pathology results) were exchanged between OVC researchers and RnaDx personnel.

Progression-free survival (PFS) time was calculated as the time from diagnosis to relapse. Statistical analyses included a linear mixed effects model and a likelihood test to assess RNA concentration and clinical response, weighted linear regression models and hypothesis tests for the relationships between RNA concentration/RDI value and neoplastic cell score. A Mann-Whitney test assessed the association of maximum RDI value with relapse timing (during or after CHOP therapy). A Cox proportional hazard model was used to assess the association of both maximum and average RDI values with PFS. Kaplan-Meier curves were generated for average and maximum RDI values and log-rank tests were used to compare median PFS.

## Supplementary information

**Additional file 1: Table A. 1.** CHOP Therapy and FNA Schedule. Drugs and dosages of CHOP therapy by week with timing of FNA collection (taken prior to treatment).

**Additional file 2: Table A. 2.** Dogs given Modified CHOP Regimen. Timing of the L-asparginase dose given as a modified CHOP regimen in four dogs and the resulting RDI values.

## Data Availability

All data generated or analysed during this study are available from the corresponding author on reasonable request and with permission of Rna Diagnostics.

## Abbreviations

CHOP: Cyclophosphamide, Doxorubicin, Vincristine and Prednisone
CR: Complete response
FNA: Fine needle aspirate
NCS: Neoplastic Cell Score
pCR: Pathological complete response
PD: Progressive disease
PFS: Progression-free survival
PR: Partial response
RDA: RNA Disruption Assay
RDI: RNA Disruption Index
RIN: RNA Integrity Number
SD: Stable disease
