# Supplementary material for: RNA disruption indicates CHOP therapy efficacy in canine lymphoma
Source: BMC Vet Res. 2019 Dec 16;15:453. doi: 10.1186/s12917-019-2189-x (PMC6916446; doi:10.1186/s12917-019-2189-x)
Supplement: Supplementary file 1 — Additional file 1: Table A. 1. CHOP Therapy and FNA Schedule. Drugs and dosages of CHOP therapy by week with timing of FNA collection (taken prior to treatment). [file 12917_2019_2189_MOESM1_ESM.docx]

**Additional File Table A.1**

**CHOP Therapy and FNA Schedule**

| **Week** | **Drugs and Dosage** | **FNA Collected for RDA** |
| --- | --- | --- |
|  |  | **(prior to treatment)** |
| **1** | vincristine 0.7 mg/m^2^ IV | yes |
|  | prednisone 2.0 mg/kg PO q 24 hr |  |
| **2** | cyclophosphamide 250 mg/m^2^ PO |  |
|  | prednisone 1.5 mg/kg PO q 24 hr |  |
| **3** | vincristine 0.7 mg/m^2^ IV | yes |
|  | prednisone 1.0 mg/kg PO q 24 hr |  |
| **4** | doxorubicin 30 mg/m^2^ IV |  |
|  | prednisone 0.5 mg/kg PO q 24 h |  |
| **6** | vincristine 0.7 mg/m^2^ IV | yes |
| **7** | cyclophosphamide 250 mg/m^2^ PO |  |
| **8** | vincristine 0.7 mg/m^2^ IV |  |
| **9** | doxorubicin 30 mg/m^2^ IV |  |
| **11** | vincristine 0.7 mg/m^2^ IV | yes |
| **13** | cyclophosphamide 250 mg/m^2^ PO |  |
| **15** | vincristine 0.7 mg/m^2^ IV |  |
| **17** | doxorubicin 30 mg/m^2^ IV |  |
| **19** | vincristine 0.7 mg/m^2^ IV | in 4 patients |
| **21** | cyclophosphamide 250 mg/m^2^ PO |  |
| **23** | vincristine 0.7 mg/m^2^ IV |  |
| **25** | doxorubicin 30 mg/m^2^ IV |  |
