# Supplementary material for: RNA disruption indicates CHOP therapy efficacy in canine lymphoma
Source: BMC Vet Res. 2019 Dec 16;15:453. doi: 10.1186/s12917-019-2189-x (PMC6916446; doi:10.1186/s12917-019-2189-x)
Supplement: Supplementary file 2 — Additional file 2: Table A. 2. Dogs given Modified CHOP Regimen. Timing of the L-asparginase dose given as a modified CHOP regimen in four dogs and the resulting RDI values. [file 12917_2019_2189_MOESM2_ESM.docx]

**Additional File Table A.2**

**Dogs given Modified CHOP Regimen**

| **Dog** | **Week of L-asparginase** | **Concurrent with Drug** | **RDA Results** | |
| --- | --- | --- | --- | --- |
|  |  |  | **Week** | **RDI Value** |
| CL1-24 | 1 | Yes | 0  2  5  11 | 0.6  0.3  3.9  3.1 |
| CL1-27 | 7 | Yes | 0  3  7  11 | 0.5  0.4  n/a  0.4 |
| CL1-28 | 0 | No; CHOP started 3 days later | 0  3  6  16 | 0.2  0.3  n/a  0.7 |
| CL1-34 | 2 | Yes | 0  4  12 | 1.3  0.6  n/a |
